# Lessons learned from a decade of immune checkpoint inhibition: The good, the bad, and the ugly

**DOI:** 10.1007/s10555-025-10260-8

**Published:** 2025-04-04

**Authors:** Jacob J. Adashek, Jillian A. Moran, Dung T. Le, Razelle Kurzrock

**Affiliations:** 1 Department of Oncology, The Johns Hopkins Hospital, The Sidney Kimmel Comprehensive Cancer Center1800 Orleans St, Baltimore, MD 21287 USA; 2 Geisel School of Medicine, Dartmouth College, 1 Rope Ferry Rd, Hanover, NH 03755 USA; 3 WIN Consortium, 24 Rue Albert Thuret, 94550 Chevilly-Larue, Paris, France; 4 University of Nebraska, 6001 Dodge St, Omaha, NE 68182 USA; 5 MCW Cancer Center, 8800 W Doyne Ave, Milwaukee, WI 53226 USA

**Keywords:** Immune checkpoint inhibition, Immune-related adverse events, Hyperprogressive disease

## Abstract

Discovering the brakes/checkpoints that cancer places on the immune system to prevent being eradicated led to the 2018 Nobel Prize and the development of multiple Food and Drug Administration-approved immune checkpoint inhibitors (ICIs). ICIs have transformed the treatment of numerous cancer types and, remarkably, some patients with end-stage metastatic disease can achieve durable, complete remissions — cures. Still, ICIs cause significant immune-related toxicities, and most tumors are resistant. Unusual progression patterns such as pseudo-progression and hyper-progression (accelerated progression) can occur. Biomarkers for ICI response/resistance include microsatellite instability, high tumor mutational burden, and PD-L1 immunohistochemistry positivity; but they are imperfect, perhaps because of immune system complexity. Herein, we explore the good, the bad, and the ugly of ICIs in cancer treatment.

## Introduction

Immune checkpoint inhibition (ICI) has revolutionized the treatment landscape for many cancers worldwide. The identification of immune checkpoints by Drs. Allison and Honjo led to their being awarded the Nobel Prize in 2018 [1] and was the impetus for the development of multiple ICI monoclonal antibodies including those that inhibit cytotoxic T-lymphocyte associated protein 4 (CTLA- 4), programmed cell death protein (ligand) 1 (PD-[L]1) and LAG- 3 [2]. The fundamental premise behind ICIs is that immune checkpoints are exploited by the tumor to inactivate the immune system, hence allowing the survival and growth of the cancer. Suppressing these checkpoints reactivates the immune system and allows it to attack the malignancy.

There are multiple Food and Drug Administration (FDA) approved ICIs (Table 1). There are also more than 35 FDA-approved combination immunotherapies for advanced cancers [3]. The extraordinary impact ICIs have had on metastatic cancers has led to the successful deployment of these drugs in earlier lines of therapy, i.e., in the neoadjuvant and adjuvant setting [4–6]. Notably, the use of ICIs in some settings has even enabled patients with widely metastatic cancers, albeit a minority, to attain durable, complete remissions, and these individuals may be cured [7].

**Table 1:** Current list of FDA-approved immune checkpoint inhibitors and their targets spanning from 2011–2024. Examples of current FDA-approved immune checkpoint inhibitors

Drug	Target*	Year approved	Comment
Atezolizumab	PD-L1	2016	
Avelumab	PD-L1	2017	
Cemiplimab	PD- 1	2018	
Dostarlimab	PD- 1	2021	Tissue-agnostic FDA approval for MSI-H/MMRd solid cancers
Durvalumab	PD-L1	2017	
Ipilimumab	CTLA- 4	2011	
Nivolumab	PD- 1	2014	
Pembrolizumab	PD- 1	2014	Two tissue-agnostic approvals: for MSI-H/MMRd and for TMB ≥ 10 mut/mb solid tumors
Relatlimab	LAG- 3	2022	Approved together with nivolumab for melanoma
Retifanlimab	PD- 1	2023	
Tislelizumab	PD- 1	2024	
Toripalimab	PD- 1	2024	
Tremelimumab	CTLA- 4	2022	Approved together with durvalumab for hepatocellular carcinoma

^*^Targets antagonize listed receptor

*CTLA- 4*, Cytotoxic T-lymphocyte associated protein 4; *MSI-H*, Microsatellite instability-high; *MMRd*, mismatch repair deficient; *PD-[L]1*, programmed cell death protein (ligand) 1; *TMB*, tumor mutational burden

Despite the successes of ICIs, the majority of patients who receive these drugs do not respond or develop secondary resistance. Significant efforts have been made to identify biomarkers in order to ascertain which patients are most likely to achieve the greatest benefit from these ICIs [8–14]; however, no single biomarker has predicted response in the majority of patients.

Importantly, ICIs can carry a considerable toxicity profile — immune-related adverse event (irAE) — that at times do not simply resolve when stopping the offending agent. These toxicities may require hospitalization, prolonged or highly potent immunosuppression and, in some rare instances, are fatal [15]. When given in combinations, ICIs can carry a grade 3 toxicity rate > 50% [16]. The silver lining here being that the use of steroids and immunosuppression to abrogate an irAE may not negatively impact overall survival (OS) [17]. Moreover, recent studies suggest that dose reduction of ICIs may ameliorate side effects without compromising activity [18]. ICIs can also result in confusing response patterns, such as pseudo-progression, which implies the enlargement of original lesions or appearance of new lesions on imaging, perhaps due to an immune inflammatory reaction, even as the actual cancer is regressing. In some instances, the use of ICIs has been shown by multiple groups across the globe to cause a phenomenon known as ‘hyperprogressive disease [19–22]. Generally, hyperprogressive disease occurs rarely, and efforts to identify a biomarker have also been underway [20].

**Fig. 1:**
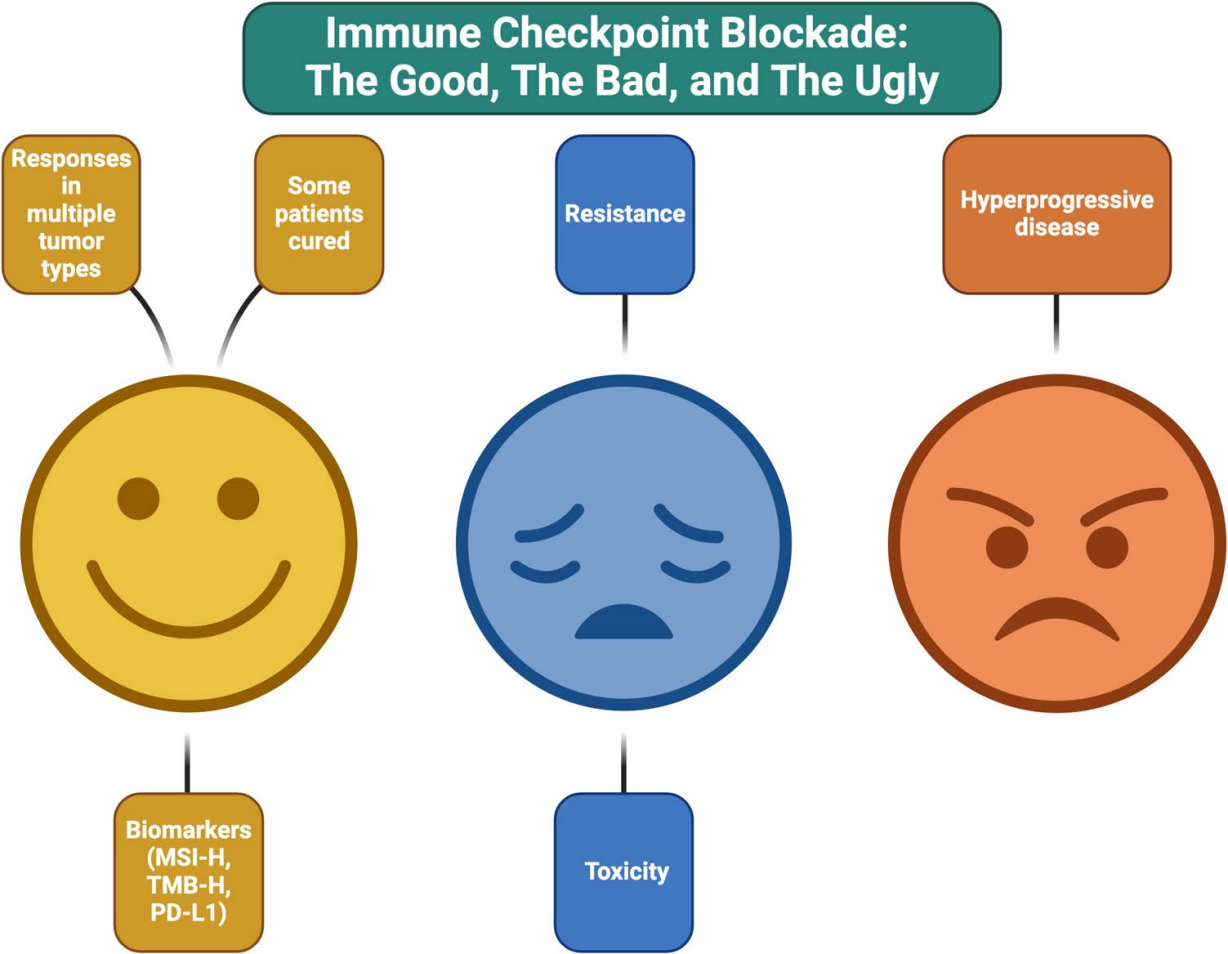
Immune checkpoint inhibitors have a complicated array of effects

Over the past decade, the use and indications of ICIs have grown exponentially. There remains a substantial amount of investigation required to identify which patients are most likely to be helped, which are most likely to be harmed, and how to harness the capability of these drugs and the immune system in order to offer patients long-lasting and meaningful responses. Herein, we provide a perspective on the good, the bad, and the ugly of ICI therapy (Fig. 1).

## The good, the bad, and the ugly

### The good

The “good” aspects of ICIs include the fact that significant subsets of a large number of different tumor types are responsive, and some of these responses are durable complete remissions, including in patients with refractory/end-stage metastatic disease. In addition, there are biomarkers, albeit imperfect, for response: microsatellite instability-high (MSI-H) or mismatch repair deficient (MMRd) and high tumor mutational burden (TMB) as well as PD-L1 positivity by immunohistochemistry (IHC). Importantly, even toxicity has two faces in the setting of ICIs, including an association with better outcome. Finally, ICIs are not myelosuppressive, nor are they cleared by the kidneys or liver, allowing treatment of patients with bone marrow, renal or hepatic dysfunction.

#### Biomarkers for response

ICIs have been remarkably beneficial for a multitude of patients with lethal metastatic cancers, even offering some complete remissions that have lasted years and are generally accepted as, “cures” [23–25]. To aid oncologists in identifying which patients may derive the most benefit from using ICIs when multiple treatment options are available, various biomarkers have been identified (Table 2).

**Table 2:** Examples of immune checkpoint inhibitor biomarkers

Biomarker	Examples of malignancies in which they appear	Examples of drugs that target the alterations
Microsatellite instability/deficient mismatch repair	Colorectal and endometrial cancer Across tumors	Pembrolizumab And dostarlimab (Both have solid cancer tissue = agnostic approval)
High tumor mutational burden ≥ 10 mutations/mb	Melanoma Across tumors	Pembrolizumab (Solid cancer tissue-agnostic approval)
PD-L1 by immunohistochemistry	Multiple FDA approvals of immune checkpoint inhibitors linked to a specific PD-L1 threshold and companion diagnostic including but not limited to bladder cancer, non-small cell lung cancer, triple-negative breast cancer, cervical cancer, and gastric/gastroesophageal junction cancer PD-L1 thresholds vary	

The use of PD-L1 IHC has been utilized as a biomarker for response to ICIs in multiple cancers [9]. Especially for PD-(L)1 inhibitors, using a marker that is directly antagonized is intuitive and clinically meaningful for many patients. Another validated biomarker for ICIs is MSI-H/MMRd [12, 13]. In a landmark study, the use of pembrolizumab led to a 40% objective response rate (ORR) in patients with MMRd colorectal cancer and 71% for non-colorectal cancers (ampullary, cholangiocarcinoma, endometrial, small bowel, gastric) [13]. The FDA, therefore, gave the first ever tumor-agnostic approval to the anti-PD- 1 pembrolizumab (in May 2017) for patients with MMRd or MSI-H solid cancers [26]. The validation of MMRd or MSI-H as biomarkers for ICI responses has also led to the solid tissue-agnostic approval of the anti-PD- 1 dostarlimab in MMRd/MSI-H cancers, which yielded a similar 44% ORR in its study [27]. With the tumor-agnostic approval of pembrolizumab, the paradigm for FDA authorization was shattered, and the era of tissue-agnostic, biomarker-based drugs was born.

It is likely that MSI-H/MMRd creates multiple mutations and hence a plethora of neo-antigens. Consequently, when an ICI reawakens the immune system, the sheer number of neo-antigens makes the likelihood that some will be immunogenic and recognized for eradication by the reactivated immune system more likely [28]. Till, while MMRd or MSI-H are valid biomarkers for responses to ICIs, they have their limitations, and some patients still do not respond.

High TMB is also a well-recognized biomarker for ICI response [8, 14]. There are various ways of calculating TMB and harmonization of methodologies may be helpful. The FDA has approved pembrolizumab for solid tumors with TMB ≥ 10 mut/Mb based on an analysis of multiple pan-cancer cohorts that reported a combined ORR of 29% [29]. Other studies have suggested different cut-offs. For instance, a TMB of ≥ 16 mut/Mb showed an ORR of 38% among diverse cancers treated with the anti-PD-L1 atezolizumab, while only 2% of patients responded if the TMB was between 10–15 mutations/mb, perhaps explaining occasional discrepant results for TMB as a biomarker when a cut off of 10 mutations/mb is utilized [30, 14].

#### Toxicity: a two-faced issue

Interestingly, patients who suffer from low-grade irAEs may have a survival advantage [31, 32]. It has been postulated that ICI-induced immune system activation can have two faces, both killing tumor cells and ‘misfiring’ on normal tissue resulting in collateral damage. Importantly, oncologists have identified potent antidotes to combat the many irAEs, including steroids, TNF-alpha inhibitors, and other immune-modulating agents [15, 33].

#### ICIs are not restricted in patients with renal, hepatic, or bone marrow dysfunction

Almost all chemotherapeutics are metabolized and excreted via the kidney and/or liver [34]. ICIs, however, are not metabolized/excreted via the kidney and/or liver and instead are broken down by proteolytic catabolism within the serum and tissues; ICIs are also not myelosuppressive [35]. These facts allow for virtually any patient to be ‘treatment-eligible’ from a standard laboratory assessment standpoint. Oncologists are also safely administering ICIs to patients who may be deemed frail or elderly [36]. Therefore, many patients who might have been deemed ineligible for more traditional chemotherapies can receive immunotherapy.

### The bad

ICIs have limitations and disappointments. First, it is important to be cognizant of the fact that although we have biomarkers for outcome, the most patients with these biomarkers still do not respond. Consequently, many patients are receiving ICI medications that are expensive and can be toxic, with no salutary effects on their cancer [37]. It has been estimated that ICIs can cost > $150,000 annually [38], though, of course, the latter is really only a problem if the patient is responding, but needs prolonged ICI therapy in order to maximize their response. Secondary resistance after initial response to ICIs is also all too common. Immune-related toxicities are frequent, and some can last a lifetime. Finally, ascertaining response can be difficult as ICIs may have unique response patterns such as pseudo-progression, wherein the tumor appears larger because of the inflammatory effect of immune infiltration but, with time, imaging will show shrinkage.

#### Imperfect biomarkers and resistance mechanisms

Though biomarkers such as MSI-H or TMB-H, as well as positive PD-L1 IHC, are important predictors of responsiveness to ICIs, they are imperfect and identify only a subset of responders [9, 8]. MSI-H results in higher TMB, which in turn results in more neo-antigens, increasing chances for T cell recognition. Even so, a composite predictor is needed; it should include critical variables such as the ability of the patient’s major histocompatibility complex (MHC) to present specific neo-antigens, the host’s T cell receptor recognition repertoire, the specific checkpoints utilized by the tumor to shield itself from the immune system (e.g. CTLA- 4, LAG- 3, etc.), and the immunogenicity of the neo-antigens produced by the mutanome [39, 14, 10]. All of these variables may underly resistance mechanisms [40]. Specific mutations have also been suggested as predictors of resistance though, in some cases, these may be simply markers of poor prognosis, regardless of the type of therapy; the confounding of prognostic and predictive markers needs to be addressed [41].

In regard to PD-L1 IHC as a biomarker of response, there may be variability in cut offs for IHC positivity for PD-L1, which can be confusing. For example, for patients with metastatic triple-negative breast cancer receiving pembrolizumab, their tumors must have a combined positive score ([CPS] (the number of PD-L1–staining tumor cells, lymphocytes, and macrophages, divided by the total number of viable tumor cells, multiplied by 100) of at least 10 [42]. Similarly, for patients with metastatic non-small cell lung cancer (NSCLC) that are being considered for treatment with the anti-PD- 1 cemiplimab, their tumor must have a tumor proportion score ([TPS] (percentage of viable tumor cells showing partial or complete membrane staining at any intensity) of at least 50% [43]. For the use of pembrolizumab in patients with metastatic NSCLC, the initial TPS IHC score requirement was ≥ 50%, but the FDA lowered the threshold down to ≥ 1% [44, 45]. These examples show the utility of PD-L1 as a biomarker for patient selection for ICIs; however, the ranges for implied benefit that the FDA allows for approval are starkly different among tumor types. Moreover, not all studies show that PD-L1 IHC is predictive; indeed, some studies suggest that PD- 1 positivity on tumor infiltrating lymphocytes (TILS) may be a more important response predictor [10].

#### Immune-related adverse events (irAEs) including lifelong toxicities

ICIs break the immune balance of the body and dampen T-cell tolerance, leading to a wide variety of immune-related toxicities, potentially affecting almost any body part. The primary treatment for immune-related adverse events (irAEs) is systemic corticosteroids, such as prednisone or methylprednisolone, which are usually the first-line therapy for irAEs and given based on the severity of the irAE; for more severe cases, additional immunosuppressive medications such anti-tumor necrosis agents, e.g., infliximab, or other organ-specific treatments may be required. Close patient monitoring, stopping treatment temporarily or permanently, and ICI dose adjustment may be needed.

While many irAEs can be abrogated with immunosuppressive agents such as high-dose steroids and TNF-alpha inhibitors, some are not. Particularly, irAE endocrinopathies may require lifelong replacement of various end-organ hormones: thyroxine, insulin, hydrocortisone [46].

#### Survival may be worsened by common medications

Some groups have found associations between various medications and outcomes in patients receiving ICIs, though these associations have not been prospectively validated. As an example, antibiotic use has been correlated with worse outcome after ICIs, perhaps because of a microbiome effect [47, 48]. It has also been reported that the use of proton pump inhibitors or angiotensin-converting enzyme inhibitors correlates with worse survival in patients receiving ICIs [49, 50]. As these medications are commonly administered, more research into their impact in the real-world is needed.

### The ugly

ICIs can cause serious irAE toxicities and, in some circumstances, these toxicities can be fatal. An additional ‘ugly’ recognized outcome of treatment with ICIs is accelerated progression, known as hyperprogressive disease.

#### Fatal toxicities

In rare instances (0.3–1.3% of patients treated), ICIs have led to lethal side effects, primarily due to either cardiac or neurologic toxic events or pneumonitis, hepatitis, or colitis [51]. Notably, patients with thymic epithelial tumors may be predisposed to severe immune toxicity due to defective immune tolerance mechanisms associated with these cancers; therefore, immunotherapy is considered contraindicated for thymomas outside the closely monitored setting of a clinical trial [52]. Unfortunately, for most cancers, the field lacks any definitive way to predict which patients are likely to experience these severe, life-altering or deadly side effects.

#### Hyperprogressive disease

An ‘ugly’ recognized outcome of treatment with ICIs is hyperprogressive disease, which means acceleration in the pace of progression after ICI treatment [19, 22]. Hyperprogressive disease is ugly for several reasons. First, it has no universal definition, though mainly it reflects a rapid increase in the pace of progression post-ICI compared to pre-ICI administration, with current definitions often including time-to-treatment failure < 2 months, > 50% increase in tumor burden compared with pre-immunotherapy imaging, and > twofold increase in progression pace [20]. Unfortunately, patients who suffer from hyperprogressive disease can have a change in rate of disease progression that can be a staggering 35- to 40-fold (compared to the pace immediately pre-ICI) and they often have an estimated survival of only ~ 3 months [20, 22]. There is no universally agreed on biomarker for hyperprogression, though some studies suggest that *MDM2* amplification and *EGFR* alterations put patients at risk for this phenomenon [20, 53]. Investigators have also suggested that effector PD- 1-positive T regulatory cells are increased after ICI treatment in patients demonstrating hyperprogression [54].

#### Pseudoprogression: A potentially confusing phenomenon

Pseudoprogression is a unique and potentially confusing phenomenon seen in a small subset of patients treated with ICIs. Pseudoprogression occurs after ICI treatment when there appears to be tumor growth on imaging studies, but this is actually due the tumors appearing larger due to immune infiltrates. Many patients with pseudopgression go on to show response. Pseudoprogression is often (but not always) accompanied with an improved general condition—the patient feels better—whereas a deteriorating general condition may indicate true progression or even hyperprogression [55]. Some studies suggest that pseudoprogression may also be distinguished from progression by monitoring blood derived cell-free DNA, with patients with the former showing a decrease in the cell-free markers and patients with progression showing an increase [56].

## Concluding remarks

Over the past decade, thousands of patients have benefited from treatment with ICIs, including durable complete responses — cures — in a subgroup with refractory end-stage metastatic cancers, proving that harnessing the immune system can be an effective strategy for eradicating malignancies. ICIs, including anti-PD- 1/PD-L1, anti-CTLA- 4 and, more recently, anti-LAG- 3 have been FDA approved in multiple tumor types. Moreover, the first compound to shatter the paradigm of drug approvals and attain a tissue-agnostic biomarker-based FDA authorization was pembrolizumab (for MSI-H/MMRd solid cancers); this approval opened the doors for multiple follow-on tissue-agnostic approvals, ushering in a new era of molecular-based drug development.

Still there are many challenges and unanswered questions associated with ICI use, including the fact that the biomarkers currently in use — MSI-H/MMRd, high TMB, and PD-L1 IHC — are imperfect at best, probably because the immune reaction to cancer is tremendously complex. Moreover, immune-related toxicities after ICIs are common, especially after combination ICI therapy, and some of these toxicities, albeit a minority, can be lifelong or fatal. Finally, the response and resistance trajectory after ICIs may be distinct from those of other therapies and include confusing phenomenon such as pseudo-progression and hyper-progression. The best time to administer ICIs is also still a matter of debate. Indeed, it is gratifying that patients with end-stage refractory cancers can respond, but a recent, albeit small, study suggests that moving therapy to newly diagnosed disease (at least in MSI-H rectal cancer) resulted in universal complete durable remissions, even permitting the patients to avoid surgery [6]. Neoadjuvant ICI therapy is also showing benefit in melanoma and squamous cell cancer of the skin [57, 58].

New checkpoint inhibitors and other immunotherapies are being developed but, unfortunately, most are in clinical trials devoid of cognate biomarkers [39, 59–62]. Hence, the immunotherapy field might do well to emulate the field of precision genomics, wherein targeted agents are now often studied in patients whose tumors harbor the molecular target [63]. Next generation precision immunotherapy clinical trials should ascertain the immune environment of each tumor and host, including but not limited to mutational burden, neo-antigen immunogenicity, ability of MHC to present neo-antigens as well as T cells to recognize them, and checkpoints exploited by the tumor. Genomic, transriptomic, proteomic and other assays can be used to interrogate the tumor for the above-mentioned characteristics and therefore select patients whose tumor immune markers best match specific immunotherapies [64].

## Data Availability

No datasets were generated or analysed during the current study.
